# Neoadjuvant chemotherapy for locally advanced gastric cancer in Japan: Consensus meeting at the 77th general meeting of the Japanese Society of Gastroenterological Surgery

**DOI:** 10.1002/ags3.12717

**Published:** 2023-07-22

**Authors:** Masanori Tokunaga, Yukinori Kurokawa, Takeo Fukagawa, Kei Muro, Kohei Shitara, Yasuhiro Kodera, Masanori Terashima

**Affiliations:** ^1^ Department of Gastrointestinal Surgery Tokyo Medical and Dental University Tokyo Japan; ^2^ Department of Gastroenterological Surgery Osaka University Graduate School of Medicine Osaka Japan; ^3^ Department of Surgery, School of Medicine Teikyo University Tokyo Japan; ^4^ Department of Clinical Oncology Aichi Cancer Center Hospital Nagoya Japan; ^5^ Department of Gastrointestinal Medical Oncology National Cancer Center Hospital East Chiba Japan; ^6^ Department of Gastroenterological Surgery Nagoya University Graduate School of Medicine Nagoya Japan; ^7^ Division of Gastric Surgery Shizuoka Cancer Center Shizuoka Japan

**Keywords:** gastric cancer, JCOG, neoadjuvant chemotherapy, perioperative chemotherapy

## Abstract

Treatment strategy for locally advanced gastric cancer differs worldwide. Neoadjuvant chemotherapy (NAC) is considered one of the promising treatment options for locally advanced gastric cancer, even in Japan, and clinical trials have been conducted or are ongoing. A consensus meeting was organized at the 77th general meeting of the Japanese Society of Gastroenterological Surgery in 2022, in which the current status and future prospects of NAC for locally advanced gastric cancer were discussed. Participants at the meeting looked forward to the results of the JCOG1509 trial, providing solid evidence regarding NAC. The optimal indications and regimens for NAC were also debated. Patients with cStage III gastric cancer are the main targets of NAC in Japan, and a doublet regimen of S‐1 and oxaliplatin was preferred by the participants. However, the feasibility of a triplet regimen with S‐1, oxaliplatin, and docetaxel, and that with 5‐FU, leucovorin, oxaliplatin, and docetaxel has been demonstrated, and these could become treatment options in Japan. Other points of discussion include perioperative chemotherapy to avoid peritoneal recurrence and for patients with dMMR/MSI‐high tumors. The panel regarded NAC as a promising treatment option, and NAC will become the standard treatment for cStage III gastric cancer in Japan if an ongoing clinical trial successfully demonstrates its efficacy.

## Graphical abstract

Discussion at a consensus meeting at the 77th general meeting of the Japanese Society of Gastroenterological Surgery in 2022, in which the current status and future prospects of NAC for locally advanced gastric cancer was summarized. NAC is a promising treatment option and will become the standard treatment of cStage IIII gastric cancer in Japan if an ongoing clinical trial successfully demonstrates its efficacy.

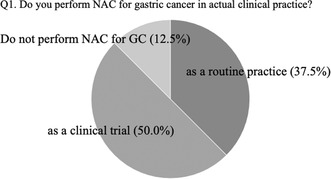

## 1 INTRODUCTION

Gastric cancer is the third leading cause of cancer‐related deaths worldwide. Different treatment strategies for locally advanced gastric cancer have been developed in Europe and East Asia.^1^ In Europe, perioperative chemotherapy has been the standard treatment since Cunningham et al.^2^ first demonstrated its efficacy in patients with locally advanced gastric cancer. On the other hand, in East Asia, postoperative adjuvant chemotherapy following D2 gastrectomy has become standard since randomized controlled trials demonstrated the efficacy of postoperative S1 and capecitabine plus oxaliplatin.^3^, ^4^, ^5^, ^6^

In Japan, the efficacy of neoadjuvant chemotherapeutic regimens continues to be investigated in the hope of improving the long‐term survival outcomes of patients with far‐advanced gastric cancer.^7^, ^8^, ^9^ At the 77th general meeting of the Japanese Society of Gastroenterological Surgery, held in Yokohama in July 2022, a consensus meeting was organized, and the current status and future prospects of neoadjuvant chemotherapy were discussed. At this meeting, past and ongoing JCOG trials, neoadjuvant chemotherapy with DOS and FLOT, and treatments for patients with dMMR/MSI‐high tumors are presented and discussed. In this review, the current status of neoadjuvant chemotherapy in Japan is summarized, and future perspectives are discussed based on presentations at that meeting.

## 2 NEOADJUVANT CHEMOTHERAPY FOR GASTRIC CANCER WITH EXPECTED POOR SURVIVAL OUTCOMES

Since adjuvant chemotherapy following D2 gastrectomy has become a standard treatment for locally advanced gastric cancer in Japan (Table 1),^3^, ^5^ the main target for NAC has been patients with locally advanced gastric cancer with an expected 5‐year survival of less than 30%.

**TABLE 1:** Trials investigating the efficacy of preoperative neoadjuvant chemotherapy (including ongoing and international trials).

Trial/authors	Phase	Patients	Arms	No. of patients	5Y‐PFS	5Y‐OS	Hazard ratio for OS (95% CI)
JCOG1509^10^	III	cStrage III	S: surgery + S‐1/DS	235^a^	Ongoing		
			E: SOX + surgery + S‐1/DS	235^a^			
PRODIGY^11^	III	T2‐3N+ or T4Nany	S: surgery + S‐1	264	60.2^b^		
			E: DOS + surgery + S‐1	266	66.3^b^		0.70 (0.52–0.95)^c^
FLOT^12^		≥T2 and/or N+	S: ECF(ECX) + surgery + ECF(ECX)	360		36%	
			E: FLOT + surgery + FLOT	356		45%	0.77 (0.63–0.94)
NAC for GC with extended LN metastasis							
JCOG0405^7^	II	Extended LN metastasis	SP + surgery	51		53%	–
JCOG1002^8^	II	Extended LN metastasis	DCS + surgery + S‐1	52		55%	–
JCOG1704^13^	II	Extended LN metastasis	DOS + surgery + S‐1	46	Ongoing		
NAC for schirrous type GC							
JCOG0501^9^	III	Type 4/large type 3	S: surgery + S‐1	149	48%^b^	62%^d^	
			E: SP + surgery + S‐1	151	48%^b^	61%^d^	0.92 (0.68–1.23)

Abbreviations: DCS, docetaxel + cisplatin + S‐1; DOS, docetaxel + oxaliplatin + S‐1; DS, docetaxel + S‐1; E, experimentary arm; ECF, epirubicin, cisplatin, fluorouracil; ECX, epirubicin, cisplatin, capecitabine; FLOT, 5‐fluorouracil + leucovorin + oxaliplatin + docetaxel; LN, lymph node; S, standard arm; SOX, S‐1 + oxaliplatin; SP, S‐1 + cisplatin.

^a^ Planed number of patients recruited for the study.

^b^ 3Y‐PFS.

^c^ Hazard ratio for PFS.

^d^ 3Y‐OS.

The first target was type 4 or large (≥8 cm in maximum diameter) type 3 gastric cancer. After the completion of the phase II trial, a randomized controlled trial (JCOG0501) was conducted. JCOG0501 was designed to demonstrate a 10% increase in the 3‐year overall survival rate after neoadjuvant chemotherapy with S‐1 plus cisplatin. However, this study failed to demonstrate superiority.^9^, ^14^ NAC with a more intensified triplet regimen or intraperitoneal chemotherapy is expected to demonstrate greater efficacy and is currently under investigation.

The second most common target was gastric cancer with extensive lymph node metastasis. The efficacy of neoadjuvant chemotherapy for bulky nodal metastases and/or para‐aortic nodal (PAN) metastases has been investigated in single‐arm phase II studies.^7^, ^8^, ^15^ In these studies, bulky nodal metastasis was defined as a single node ≥3.0 cm or two or more nodes ≥1.5 cm in size at the suprapancreatic area. The JCOG0405 phase II study investigated the efficacy of neoadjuvant S‐1 plus cisplatin in this population. The R0 resection rate, which was set as the primary endpoint with a threshold value of 50%, was 82% (95% confidence interval [CI], 69%–92%); therefore, the null hypothesis was rejected.^7^ Long‐term survival outcomes were far better than expected, with 3‐ and 5‐year overall survival rates of 59% and 53%, respectively. Subsequently, neoadjuvant chemotherapy with docetaxel, cisplatin, and S‐1 (DCS) was administered in JCOG1002 with the same target population. Although the clinical response rate (57.7%), which was set as the primary endpoint, was less than the threshold value of 65%, the 5‐year survival rate (55%) was similar to that of the JCOG0405^8^ trial. After JCOG1002, another phase II study (JCOG1704) was conducted, and the efficacy of neoadjuvant chemotherapy with docetaxel, oxaliplatin, and S‐1 (DOS) in patients with gastric cancer and extensive nodal metastasis was evaluated, with the primary endpoint being the major pathological response rate.^13^

## 3 NEOADJUVANT CHEMOTHERAPY FOR STAGE III GASTRIC CANCER IN JAPAN

As the survival outcomes of patients with pStage III gastric cancer are still dismal even with postoperative adjuvant chemotherapy, more intensified perioperative chemotherapy is warranted, and NAC is deemed a plausible option.^16^, ^17^ To elucidate optimal candidates for NAC, a prospective cross‐sectional study, JCOG1302A, was conducted.^18^ In JCOG1302A, 1250 patients with ≥cT2 gastric cancer were included, and the concordance rate between clinical and pathological stages was assessed. Based on the results of JCOG1302A, ≥cT4N+, equivalent to cStage III, was selected as the optimal inclusion criteria for a subsequent clinical trial, with a contamination rate of pStage I being 6.5% and sensitivity of pStage III being 64.5%.

The JCOG1509 trial was a randomized controlled trial aimed at confirming the efficacy of neoadjuvant chemotherapy with S‐1 and oxaliplatin in patients with cStage III gastric cancer. A total of 470 patients are expected to be enrolled by March 2025.^10^

In most clinical trials, other than JCOG1002 and JCOG1704, doublet regimens with S‐1 plus cisplatin/oxaliplatin were adopted as platform regimens; however, neoadjuvant chemotherapy with a triplet regimen is standard in Europe and has also been investigated in clinical trials in East Asia.^11^, ^19^

## 4 NEOADJUVANT CHEMOTHERAPY WITH DOS

The efficacy of neoadjuvant chemotherapy with DOS was investigated in the PRODIGY study conducted in Korea.^11^ In PRODIGY, progression‐free survival was set as the primary endpoint, and 530 patients with cT3‐4Nany locally advanced gastric cancer were allocated to either the CSC group (neoadjuvant chemotherapy with DOS before D2 gastrectomy followed by adjuvant S‐1; *n* = 266) or the SC group (D2 gastrectomy followed by adjuvant S‐1; *n* = 264). In the CSC group, docetaxel (50 mg/m^2^) and oxaliplatin (100 mg/m^2^) were administered intravenously on day 1, and S1 (40 mg/m^2^) was administered orally twice daily from days 1 to 14. These treatments were repeated every 3 weeks for three courses. Grade 3 or higher adverse events observed during neoadjuvant chemotherapy included neutropenia (12.6%), febrile neutropenia (9.2%), and diarrhea (5.0%). Progression‐free survival was better in the CSC group than in the CS group, with an adjusted hazard ratio of 0.70 (95% CI, 0.52–0.95; stratified log‐rank *p* = 0.023).

The feasibility of neoadjuvant chemotherapy with DOS has been investigated in Japan. Kurokawa et al.^20^ conducted a phase II trial on neoadjuvant DOS in patients with clinical stage III gastric or esophagogastric junction adenocarcinoma. Fifty patients were recruited and received two or three courses, 3‐week cycles of docetaxel (40 mg/m^2^) and oxaliplatin (100 mg/m^2^) on day 1, plus oral S‐1 (40 mg/m^2^ twice a day) from days 1 to 14. Grade 3–4 neutropenia and diarrhea were observed in 69% and 19% of patients, respectively. R0 resection was achieved in 92% of the patients, and ≥ grade Ib pathological response according to the Japanese Classification of Gastric Carcinoma (JCGC) was observed in 63% of the patients.

Saito et al.^21^ focused on the efficacy of neoadjuvant DOS against cStage IIB–IV adenocarcinoma of the esophagogastric junction (Siewert types I–III). In their retrospective study that included 36 patients, 28 (78%) patients received three or more 3‐week cycles of docetaxel (40 mg/m^2^) and oxaliplatin (100 mg/m^2^) on day 1, plus oral S‐1 (40 mg/m^2^ twice a day) from days 1 to 14. During neoadjuvant chemotherapy, grade 3–4 neutropenia, febrile neutropenia, anorexia, and diarrhea were observed in 26 (72%), 6 (17%), 7 (19%), and 4 (11%) patients, respectively. Pathological complete response was observed in 11 (31%) patients, and ≥grade 2 pathological response according to the JCGC was observed in 17 (47%) patients.

## 5 NEOADJUVANT CHEMOTHERAPY WITH 5‐FU, LEUCOVORIN, OXALIPLATIN, AND DOCETAXEL (FLOT)

In Europe, the FLOT regimen is one of the standard first‐line chemotherapeutic regimens for patients with unresectable/advanced gastric cancer.^22^ The efficacy of FLOT as a perioperative chemotherapeutic regimen has also been investigated in FLOT4.^12^ In that study, the superiority of neoadjuvant FLOT over conventional neoadjuvant ECX/EOX was demonstrated, with a hazard ratio for OS being 0.77. The number of treatment‐related severe adverse events and deaths was similar between the treatment arms. Accordingly, FLOT has become the standard perioperative chemotherapeutic regimen in Europe.

## 6 PERIOPERATIVE CHEMOTHERAPY AIMING TO PREVENT PERITONEAL METASTASIS

Peritoneal metastasis is one of the most frequently observed recurrence patterns following curative gastrectomy, accounting for 44.3% of all recurrences, according to the Japanese nationwide database.^23^ Postoperative adjuvant chemotherapy is expected to decrease the incidence of peritoneal metastasis, but no regimen has been reported to significantly decrease peritoneal metastasis.^3^, ^5^, ^17^

JCOG0501 is a randomized controlled trial investigating the efficacy of preoperative SP in patients with type 4 or large type 3 advanced gastric cancer. This study included patients with localized peritoneal metastasis and/or positive lavage cytology, as R0/R1 resection may be possible. Peritoneal metastasis was most frequently observed, accounting for 78% of all recurrences; however, preoperative SP did not decrease the incidence of peritoneal metastasis or improve survival outcomes.^9^, ^24^

Intraperitoneal chemotherapy is another approach for treating peritoneal metastases. PHOENIX‐GC is a randomized controlled trial that aims to demonstrate the superiority of intraperitoneal and intravenous paclitaxel plus S‐1 over intravenous cisplatin plus S‐1 in patients with gastric cancer with peritoneal metastasis.^25^ Although the study failed to demonstrate superior overall survival, which was set as a primary endpoint, sensitivity analysis adjusted for baseline ascites did demonstrate better overall survival with intraperitoneal chemotherapy with a hazard ratio of 0.59 (95% CI, 0.39–0.87).

The efficacy of perioperative intraperitoneal chemotherapy has been evaluated in patients at high risk of peritoneal recurrence. The INPACT was a randomized phase II trial that investigated the superiority of intraperitoneal paclitaxel over intravenous paclitaxel.^26^ Patients with resectable gastric linitis plastica, cancer with a minimal number of peritoneal deposits, or cancer with positive lavage cytology were included, and intraperitoneal or intravenous paclitaxel was administered following gastrectomy. In the INPACT trial, the 2‐year overall survival rate was set as the primary endpoint; however, the superiority of intraperitoneal paclitaxel over intravenous paclitaxel has not been proven.

Recently, Ishigami et al.^27^ commenced the PHOENIX‐GC2 trial, in which patients with type 4 gastric cancer without apparent distant or peritoneal metastasis were included and randomized based on peritoneal lavage cytology findings. When the result of peritoneal lavage cytology is negative, the patients will undergo upfront gastrectomy and will be randomized to receive either postoperative adjuvant chemotherapy with S‐1 plus intravenous docetaxel or adjuvant chemotherapy with S‐1 plus intravenous and intraperitoneal paclitaxel. When the result of peritoneal lavage cytology is positive, patients will be randomized into either the gastrectomy with perioperative systemic chemotherapy group or the gastrectomy with perioperative systemic and intraperitoneal chemotherapy group.

Hyperthermic intraperitoneal chemotherapy (HIPEC), first reported in Japan, is another option for preventing peritoneal recurrence.^28^, ^29^ However, because HIPEC is not widely accepted as the standard of care in Japan, its efficacy has been investigated mostly in Europe. Clinical trials are ongoing, and HIPEC may become the standard treatment for patients with a high probability of peritoneal metastasis.^30^, ^31^, ^32^

## 7 PERIOPERATIVE CHEMOTHERAPY FOR dMMR/MSI‐H GASTRIC CANCER

Although dMMR and microsatellite instability (MSI)‐high (H) have been recognized as strong biomarkers for predicting the sensitivity to immune checkpoint inhibitors, no phase III study has focused on the efficacy of perioperative immune checkpoint inhibitors in patients with dMMR/MSI‐H gastric cancer, probably because of the limited number of cases. However, the possibility of detrimental effects of conventional perioperative chemotherapy in dMMR/MSI‐H gastric cancer has been reported in a meta‐analysis.^33^

This meta‐analysis included individual data from four randomized controlled trials investigating the efficacy of perioperative chemotherapy in patients with locally advanced gastric cancer and demonstrated the detrimental effects of perioperative chemotherapy in MSI‐H patients.^2^, ^4^, ^33^, ^34^, ^35^ In this study, patients with MSI‐low or microsatellite‐stable (MSS) disease benefited from chemotherapy, whereas those with MSI‐H did not. The authors concluded that chemotherapy omission and/or immune checkpoint blockade should be investigated for MSI‐H gastric cancer.

The chemosensitivity of dMMR/MSI‐high gastric cancer was also explored in KEYNOTE‐062, which investigated the superiority of chemotherapy plus pembrolizumab and the non‐inferiority of pembrolizumab monotherapy over conventional chemotherapy in patients with unresectable/recurrent gastric cancer with a programmed cell death ligand 1 (PD‐L1) combined positive score (CPS) of 1 or greater.^36^ An exploratory analysis of KEYNOTE‐062 data looking only at MSI‐H patients found that both pembrolizumab and pembrolizumab plus chemotherapy were superior to chemotherapy alone, with hazard ratios of 0.29 and 0.37, respectively, indicating that pembrolizumab monotherapy may be as effective as pembrolizumab plus chemotherapy.

Similar results were confirmed in CheckMate‐649, which evaluated the treatment of metastatic gastric cancer with the PD‐1 antibody nivolumab and the CTLA‐4 antibody ipilimumab. In MSI‐High with any CPS population, survival outcomes of nivolumab plus chemothapy group and nivolumab plus imilimumab group were better than that of chemotherapy group with hazard rations of 0.38 and 0.28, respectively.^37^

A phase II trial (NEONIPIGA) of neoadjuvant nivolumab and ipilimumab in resectable gastric cancer with dMMR/MSI‐H showed pathological CR (pCR) in 19 (58.8%) of 29 patients who underwent R0 resection. No unexpected toxicity or safety issues were observed. These findings raise expectations for preoperative MMR/MSI status as a strong biomarked and, in the case of dMMR or MSI‐H, preoperative treatment with immunotherapy alone or in combination with immunotherapy and chemotherapy may be sufficient. In the future, it may be possible to perform a watch‐and‐wait procedure, as in operable rectal cancer, and preserve the stomach without surgery. We look forward to future clinical trials and developments.^38^

## 8 REAL‐WORLD PRACTICE OF NEOADJUVANT CHEMOTHERAPY IN JAPAN

At the end of the consensus meeting, several questions regarding neoadjuvant chemotherapy were displayed on the screen, and all participants in the session answered each question using an answer pad.

**FIGURE 1:**
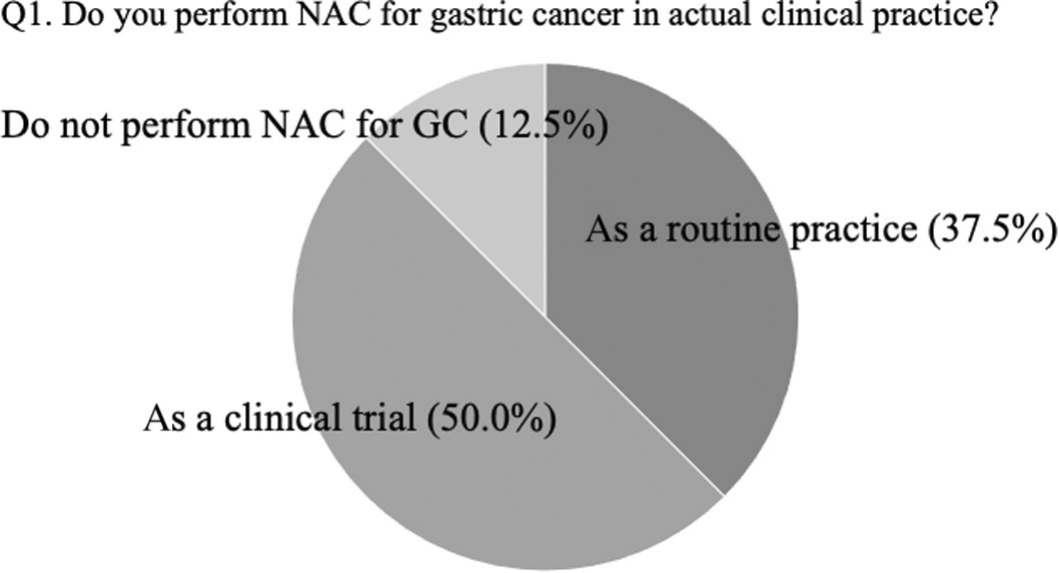
The answers to question 1 at the consensus meeting: do you perform NAC for gastric cancer in actual clinical practice?

The first question asked whether participants performed neoadjuvant chemotherapy for gastric cancer in actual practice; 37.5% answered that they did perform neoadjuvant chemotherapy as a routine practice, while 50% reported that they performed it when participating in clinical trials (Figure 1). The remaining 12.5% of participants answered that they did not perform neoadjuvant chemotherapy for gastric cancer.

**FIGURE 2:**
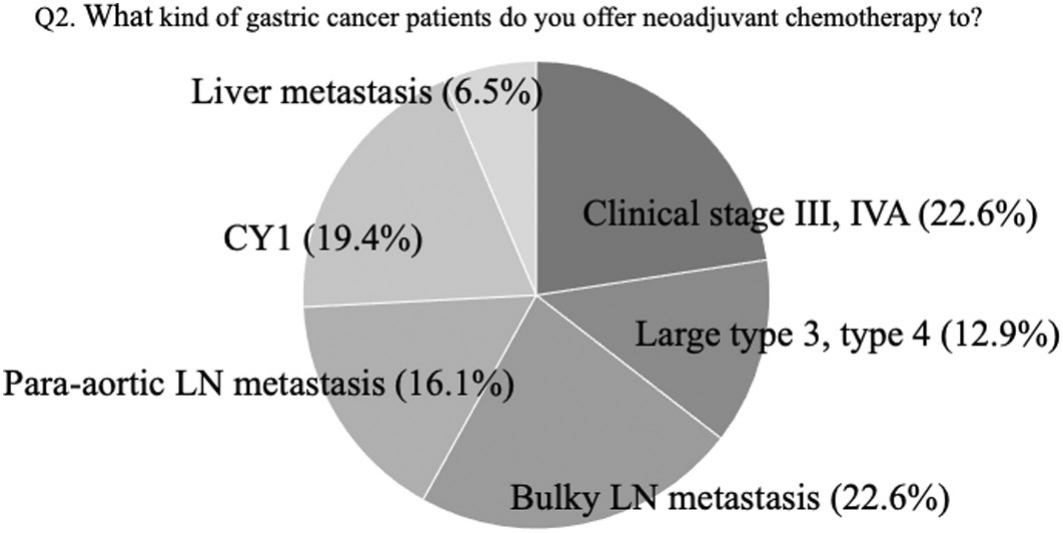
The answers to question 2 at the consensus meeting: what kind of gastric cancer patients do you offer neoadjuvant chemotherapy to (multiple answers are allowed)? CY1, positive lavage cytology; LN, lymph node.

The second question asked what types of gastric cancer patients were offered neoadjuvant chemotherapy. Multiple answers were provided for this question (Figure 2). Gastric cancer patients with clinical stage III or IVA disease, bulky lymph node metastasis, and positive cytology results were selected as candidates for neoadjuvant chemotherapy.

**FIGURE 3:**
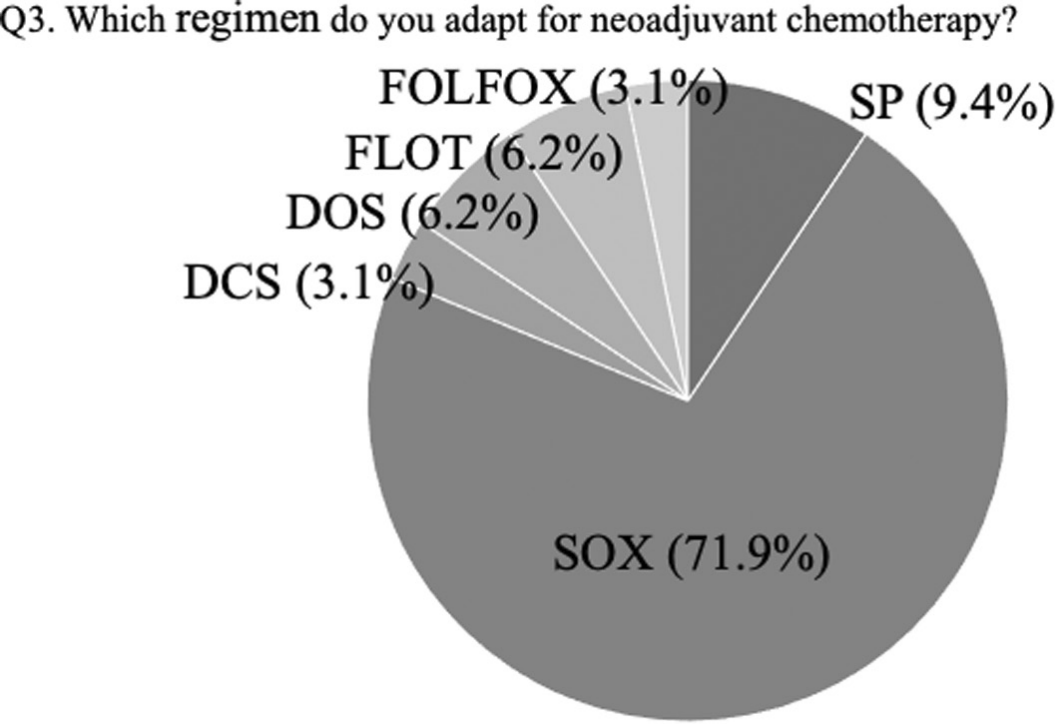
The answers to question 3 at the consensus meeting: which regimen do you adapt for neoadjuvant chemotherapy? DCS, docetaxel, cisplatin, plus S‐1; DOS, docetaxel, oxaliplatin, plus S‐1; FLOT, 5‐FU, leucovorin, plus docetaxel; FOLFOX, leucovorin, 5‐FU, plus oxaliplatin; SOX, S‐1 plus oxaliplatin; SP, S‐1 plus cisplatin.

The last question asked which regimen they chose as neoadjuvant chemotherapy (Figure 3). Most participants (71.9%) selected the SOX regimen for neoadjuvant chemotherapy, while 15.5% as FOLFOX is not a triplet regimen, such as DCS, DOS, or FLOT.

## 9 DISCUSSION

The efficacy of neoadjuvant chemotherapy has been evaluated and promising results have been reported not only in Europe but also in Asia. Accordingly, NAC is expected to become the standard treatment for locally advanced gastric cancer even in Asia.^2^, ^11^, ^19^ In total, 87.5% of the participants at the consensus meeting stated that they had undergone NAC as part of a clinical trial (37.5%) or in daily practice (50.0%). NAC is also expected to become a standard treatment in Japan; however, the final results of an ongoing clinical trial should be awaited.^10^

The optimal indication criteria and most suitable regimens for NAC are still debated and may differ among countries. In Europe, where a screening system for gastric cancer has not been established and the majority of cases are diagnosed at an advanced stage, ≥cStage IB could be an optimal indication for NAC, as recommended in the ESMO guidelines.^39^ However, in Japan, a well‐established screening system enables surgeons/oncologists to diagnose gastric cancer at an earlier stage. Accordingly, if NAC is administered to all patients with ≥cStage IB gastric cancer, many pStage I patients, whose expected 5‐year survival rate is >90% without perioperative chemotherapy, may be contaminated and overtreated. Therefore, the main targets for neoadjuvant chemotherapy had been patients with far advanced disease, such as type 4 gastric cancer or gastric cancer with bulky node/PAN metastasis, with expected dismal long‐term survival outcomes.^7^, ^8^, ^9^, ^14^, ^15^ However, JCOG1302A successfully demonstrated that gastric cancer patients with cStage III disease could be optimal candidates for NAC in Japan, with an acceptable contamination rate of pStage I. Recent Asian clinical trials investigating NAC, as well as JCOG1509, have adopted similar or the same inclusion criteria.

The optimal NAC regimens may differ among countries. In Europe, where most candidates have marginally resectable gastric cancer, a more intensified triplet regimen should be selected, despite severe toxicity. However, most cStage III gastric cancers in Japan are technically resectable; therefore, the NAC regimen should be less toxic so that gastrectomy can be performed as scheduled. Indeed, 71.9% of participants in the consensus meeting selected SOX as the preferred NAC regimen. The efficacy of NAC with SOX is under investigation in JCOG1509, and we await the results before NAC can be accepted as a standard treatment for cStage III gastric cancer.^10^, ^11^, ^19^

The latest Japanese Gastric Cancer Treatment Guidelines recommend nivolumab for HER2 negative and trastuzumab for HER2 positive unresectable/recurrent gastric cancer as first‐line chemotherapy.^40^ Theoretically, NAC regimens should be selected based on HER2 status, and the addition of nivolumab or trastuzumab may accelerate the efficacy of NAC. However, this needs to be investigated and clarified in future studies.

NAC is sometimes confused with conversion surgery, and the differences in definitions should be shared among surgical and medical oncologists. Recently, Yoshida et al. defined conversion surgery as a surgical treatment aimed at R0 resection after chemotherapy for tumors that were originally regarded as technically or oncologically unresectable or only marginally resectable.^41^, ^42^ According to this definition, preoperative chemotherapy for patients with PAN or liver metastasis deemed unresectable before chemotherapy should not be regarded as NAC.

The current status and future perspectives of NAC for locally advanced gastric cancer were presented and discussed at the consensus meeting of the 7th general meeting of the Japanese Society of Gastroenterological Surgery. The presenters and participants regarded NAC as a promising treatment option, and NAC will become the standard treatment for cStage III gastric cancer in Japan if an ongoing clinical trial successfully demonstrates its efficacy.

## CONFLICT OF INTEREST STATEMENT

M. Tokunaga has received lecture fees from Ono Pharmaceutical, Lilly, and Daiichi Sankyo. Y. Kurokawa is an Associate Editor of *Annals of Gastroenterological Surgery*, has received lecture fees from Yakult Honsha and Taiho Pharmaceutical, and has received research expense from Yakult Honsha and Taiho Pharmaceutical. K. Muro has received lecture fees from Lilly, Daiichi Sankyo, Ono Pharmaceutical, Taiho Pharmaceutical, Takeda, and Bristol‐Myers Squibb, and has received research expenses from Sanofi, Eisai, Astellas, Amgen, Daiichi Sankyo, Novartis, Taiho Pharmaceutical, MSD, Ono pharmaceutical, and Chugai. K. Shitara is advisor position of Lilly, Bristol Myers Squibb, Takeda, Pfizer, Ono Pharmaceutical, Merck Pharmaceutical, Taiho Pharmaceutical, Astellas, Novartis, AbbVie, GlaxoSmithKline, Daiichi Sankyo, Amgen, Boehringer Ingelheim, Guardant Health Japan, and Janssen, has received lecture fees from Takeda, Bristol‐Myers Squibb and Janssen, and has received research expenses from Astellas, Ono Pharmaceutical, Daiichi Sankyo, Taiho Pharmaceutical, Chugai, Merck Pharmaceutical, Medi Science, Eisai and Amgen. Y. Kodera is an Associate Editor of *Annals of Gastroenterological Surgery*, and has lecture fees from Taiho Pharmaceutical and Chugai, and has received research expenses from Taiho Pharmaceutical and Chugai. All remaining authors declare no conflicts of interest.

## ETHICS STATEMENT

Approval of the research protocol: N/A.

Informed Consent: N/A.

Registry and the Registration No. of the study/trial: N/A.

Animal Studies: N/A.

## References

[1] Bray F , Ferlay J , Soerjomataram I , Siegel RL , Torre LA , Jemal A . Global cancer statistics 2018: GLOBOCAN estimates of incidence and mortality worldwide for 36 cancers in 185 countries. CA Cancer J Clin. 2018;68(6):394–424. 30207593 10.3322/caac.21492

[2] Cunningham D , Allum WH , Stenning SP , Thompson JN , van de Velde C , Nicolson M , et al. Perioperative chemotherapy versus surgery alone for resectable gastroesophageal cancer. N Engl J Med. 2006;355(1):11–20. 16822992 10.1056/NEJMoa055531

[3] Sakuramoto S , Sasako M , Yamaguchi T , Kinoshita T , Fujii M , Nashimoto A , et al. Adjuvant chemotherapy for gastric cancer with S‐1, an oral fluoropyrimidine. N Engl J Med. 2007;357(18):1810–1820. 17978289 10.1056/NEJMoa072252

[4] Bang YJ , Kim YW , Yang HK , Chung HC , Park YK , Lee KH , et al. Adjuvant capecitabine and oxaliplatin for gastric cancer after D2 gastrectomy (CLASSIC): a phase 3 open‐label, randomised controlled trial. Lancet. 2012;379(9813):315–321. 22226517 10.1016/S0140-6736(11)61873-4

[5] Sasako M , Sakuramoto S , Katai H , Kinoshita T , Furukawa H , Yamaguchi T , et al. Five‐year outcomes of a randomized phase III trial comparing adjuvant chemotherapy with S‐1 versus surgery alone in stage II or III gastric cancer. J Clin Oncol. 2011;29(33):4387–4393. 22010012 10.1200/JCO.2011.36.5908

[6] Noh SH , Park SR , Yang HK , Chung HC , Chung IJ , Kim SW , et al. Adjuvant capecitabine plus oxaliplatin for gastric cancer after D2 gastrectomy (CLASSIC): 5‐year follow‐up of an open‐label, randomised phase 3 trial. Lancet Oncol. 2014;15(12):1389–1396. 25439693 10.1016/S1470-2045(14)70473-5

[7] Tsuburaya A , Mizusawa J , Tanaka Y , Fukushima N , Nashimoto A , Sasako M , et al. Neoadjuvant chemotherapy with S‐1 and cisplatin followed by D2 gastrectomy with para‐aortic lymph node dissection for gastric cancer with extensive lymph node metastasis. Br J Surg. 2014;101(6):653–660. 24668391 10.1002/bjs.9484

[8] Ito S , Sano T , Mizusawa J , Takahari D , Katayama H , Katai H , et al. A phase II study of preoperative chemotherapy with docetaxel, cisplatin, and S‐1 followed by gastrectomy with D2 plus para‐aortic lymph node dissection for gastric cancer with extensive lymph node metastasis: JCOG1002. Gastric Cancer. 2017;20(2):322–331. 27299887 10.1007/s10120-016-0619-z

[9] Iwasaki Y , Terashima M , Mizusawa J , Katayama H , Nakamura K , Katai H , et al. Gastrectomy with or without neoadjuvant S‐1 plus cisplatin for type 4 or large type 3 gastric cancer (JCOG0501): an open‐label, phase 3, randomized controlled trial. Gastric Cancer. 2021;24(2):492–502. 33200303 10.1007/s10120-020-01136-7

[10] Terashima M , Yoshikawa T , Boku N , Ito S , Tsuburaya A , Iwasaki Y , et al. Current status of perioperative chemotherapy for locally advanced gastric cancer and JCOG perspectives. Jpn J Clin Oncol. 2020;50(5):528–534. 32134452 10.1093/jjco/hyaa005

[11] Kang YK , Yook JH , Park YK , Lee JS , Kim YW , Kim JY , et al. PRODIGY: a phase III study of neoadjuvant docetaxel, oxaliplatin, and S‐1 plus surgery and adjuvant S‐1 versus surgery and adjuvant S‐1 for resectable advanced gastric cancer. J Clin Oncol. 2021;39(26):2903–2913. 34133211 10.1200/JCO.20.02914 PMC8425847

[12] Al‐Batran SE , Homann N , Pauligk C , Goetze TO , Meiler J , Kasper S , et al. Perioperative chemotherapy with fluorouracil plus leucovorin, oxaliplatin, and docetaxel versus fluorouracil or capecitabine plus cisplatin and epirubicin for locally advanced, resectable gastric or gastro‐oesophageal junction adenocarcinoma (FLOT4): a randomised, phase 2/3 trial. Lancet. 2019;393(10184):1948–1957. 30982686 10.1016/S0140-6736(18)32557-1

[13] Sato Y , Kurokawa Y , Doki Y , Mizusawa J , Tanaka K , Katayama H , et al. A phase II study of preoperative chemotherapy with docetaxel, oxaliplatin and S‐1 in gastric cancer with extensive lymph node metastasis (JCOG1704). Future Oncol. 2020;16(4):31–38. 31920105 10.2217/fon-2019-0528

[14] Iwasaki Y , Sasako M , Yamamoto S , Nakamura K , Sano T , Katai H , et al. Phase II study of preoperative chemotherapy with S‐1 and cisplatin followed by gastrectomy for clinically resectable type 4 and large type 3 gastric cancers (JCOG0210). J Surg Oncol. 2013;107(7):741–745. 23400787 10.1002/jso.23301

[15] Yoshikawa T , Sasako M , Yamamoto S , Sano T , Imamura H , Fujitani K , et al. Phase II study of neoadjuvant chemotherapy and extended surgery for locally advanced gastric cancer. Br J Surg. 2009;96(9):1015–1022. 19644974 10.1002/bjs.6665

[16] Kakeji Y , Yoshida K , Kodera Y , Kochi M , Sano T , Ichikawa W , et al. Three‐year outcomes of a randomized phase III trial comparing adjuvant chemotherapy with S‐1 plus docetaxel versus S‐1 alone in stage III gastric cancer: JACCRO GC‐07. Gastric Cancer. 2022;25(1):188–196. 34351555 10.1007/s10120-021-01224-2

[17] Yoshida K , Kodera Y , Kochi M , Ichikawa W , Kakeji Y , Sano T , et al. Addition of docetaxel to oral fluoropyrimidine improves efficacy in patients with stage III gastric cancer: interim analysis of JACCRO GC‐07, a randomized controlled trial. J Clin Oncol. 2019;37(15):1296–1304. 30925125 10.1200/JCO.18.01138 PMC6524985

[18] Fukagawa T , Katai H , Mizusawa J , Nakamura K , Sano T , Terashima M , et al. A prospective multi‐institutional validity study to evaluate the accuracy of clinical diagnosis of pathological stage III gastric cancer (JCOG1302A). Gastric Cancer. 2018;21(1):68–73. 28194522 10.1007/s10120-017-0701-1

[19] Zhang X , Liang H , Li Z , Xue Y , Wang Y , Zhou Z , et al. Perioperative or postoperative adjuvant oxaliplatin with S‐1 versus adjuvant oxaliplatin with capecitabine in patients with locally advanced gastric or gastro‐oesophageal junction adenocarcinoma undergoing D2 gastrectomy (RESOLVE): an open‐label, superiority and non‐inferiority, phase 3 randomised controlled trial. Lancet Oncol. 2021;22(8):1081–1092. 34252374 10.1016/S1470-2045(21)00297-7

[20] Kurokawa Y , Kawase T , Takeno A , Furukawa H , Yoshioka R , Saito T , et al. Phase 2 trial of neoadjuvant docetaxel, oxaliplatin, and S‐1 for clinical stage III gastric or esophagogastric junction adenocarcinoma. Ann Gastroenterol Surg. 2023;7:247–254. 36998295 10.1002/ags3.12632 PMC10043771

[21] Saito T , Kurokawa Y , Takahashi T , Yamamoto K , Yamashita K , Tanaka K , et al. Neoadjuvant docetaxel, oxaliplatin and S‐1 (DOS) combination chemotherapy for patients with resectable adenocarcinoma of esophagogastric junction. Gastric Cancer. 2022;25(5):966–972. 35488968 10.1007/s10120-022-01300-1

[22] Al‐Batran SE , Hartmann JT , Hofheinz R , Homann N , Rethwisch V , Probst S , et al. Biweekly fluorouracil, leucovorin, oxaliplatin, and docetaxel (FLOT) for patients with metastatic adenocarcinoma of the stomach or esophagogastric junction: a phase II trial of the Arbeitsgemeinschaft Internistische Onkologie. Ann Oncol. 2008;19(11):1882–1887. 18669868 10.1093/annonc/mdn403

[23] Katai H , Ishikawa T , Akazawa K , Isobe Y , Miyashiro I , Oda I , et al. Five‐year survival analysis of surgically resected gastric cancer cases in Japan: a retrospective analysis of more than 100,000 patients from the nationwide registry of the Japanese gastric cancer association (2001–2007). Gastric Cancer. 2018;21(1):144–154. 28417260 10.1007/s10120-017-0716-7

[24] Terashima M , Iwasaki Y , Mizusawa J , Katayama H , Nakamura K , Katai H , et al. Randomized phase III trial of gastrectomy with or without neoadjuvant S‐1 plus cisplatin for type 4 or large type 3 gastric cancer, the short‐term safety and surgical results: Japan Clinical Oncology Group Study (JCOG0501). Gastric Cancer. 2019;22(5):1044–1052. 30827001 10.1007/s10120-019-00941-z

[25] Ishigami H , Fujiwara Y , Fukushima R , Nashimoto A , Yabusaki H , Imano M , et al. Phase III trial comparing intraperitoneal and intravenous paclitaxel plus S‐1 versus cisplatin plus S‐1 in patients with gastric cancer with peritoneal metastasis: PHOENIX‐GC trial. J Clin Oncol. 2018;36(19):1922–1929. 29746229 10.1200/JCO.2018.77.8613

[26] Takahashi N , Kanda M , Yoshikawa T , Takiguchi N , Fujitani K , Miyamoto K , et al. A randomized phase II multicenter trial to explore efficacy of weekly intraperitoneal in comparison with intravenous paclitaxel administered immediately after gastrectomy to the patients with high risk of peritoneal recurrence: final results of the INPACT trial. Gastric Cancer. 2018;21(6):1014–1023. 29536296 10.1007/s10120-018-0817-y

[27] Ishigami H , Tsuji Y , Shinohara H , Kodera Y , Kanda M , Yabusaki H , et al. Intraperitoneal chemotherapy as adjuvant or perioperative chemotherapy for patients with type 4 scirrhous gastric cancer: PHOENIX‐GC2 trial. J Clin Med. 2021;10(23):5666. 34884367 10.3390/jcm10235666 PMC8658657

[28] Hamazoe R , Maeta M , Kaibara N . Intraperitoneal thermochemotherapy for prevention of peritoneal recurrence of gastric cancer. Final results of a randomized controlled study. Cancer. 1994;73(8):2048–2052. 8156509 10.1002/1097-0142(19940415)73:8<2048::aid-cncr2820730806>3.0.co;2-q

[29] Koga S , Hamazoe R , Maeta M , Shimizu N , Murakami A , Wakatsuki T . Prophylactic therapy for peritoneal recurrence of gastric cancer by continuous hyperthermic peritoneal perfusion with mitomycin C. Cancer. 1988;61(2):232–237. 3121165 10.1002/1097-0142(19880115)61:2<232::aid-cncr2820610205>3.0.co;2-u

[30] Glehen O , Passot G , Villeneuve L , Vaudoyer D , Bin‐Dorel S , Boschetti G , et al. GASTRICHIP: D2 resection and hyperthermic intraperitoneal chemotherapy in locally advanced gastric carcinoma: a randomized and multicenter phase III study. BMC Cancer. 2014;14:183. 24628950 10.1186/1471-2407-14-183 PMC3995601

[31] Götze TO , Piso P , Lorenzen S , Bankstahl US , Pauligk C , Elshafei M , et al. Preventive HIPEC in combination with perioperative FLOT versus FLOT alone for resectable diffuse type gastric and gastroesophageal junction type II/III adenocarcinoma – the phase III “PREVENT” – (FLOT9) trial of the AIO/CAOGI/ACO. BMC Cancer. 2021;21(1):1158. 34715810 10.1186/s12885-021-08872-8 PMC8555172

[32] van der Kaaij RT , Wassenaar ECE , Koemans WJ , Sikorska K , Grootscholten C , Los M , et al. Treatment of PERItoneal disease in stomach cancer with cytoreductive surgery and hyperthermic intraPEritoneal chemotherapy: PERISCOPE I initial results. Br J Surg. 2020;107(11):1520–1528. 32277764 10.1002/bjs.11588

[33] Pietrantonio F , Miceli R , Raimondi A , Kim YW , Kang WK , Langley RE , et al. Individual patient data meta‐analysis of the value of microsatellite instability as a biomarker in gastric cancer. J Clin Oncol. 2019;37(35):3392–3400. 31513484 10.1200/JCO.19.01124

[34] Bajetta E , Floriani I , Di Bartolomeo M , Labianca R , Falcone A , Di Costanzo F , et al. Randomized trial on adjuvant treatment with FOLFIRI followed by docetaxel and cisplatin versus 5‐fluorouracil and folinic acid for radically resected gastric cancer. Ann Oncol. 2014;25(7):1373–1378. 24728035 10.1093/annonc/mdu146

[35] Lee J , Lim DH , Kim S , Park SH , Park JO , Park YS , et al. Phase III trial comparing capecitabine plus cisplatin versus capecitabine plus cisplatin with concurrent capecitabine radiotherapy in completely resected gastric cancer with D2 lymph node dissection: the ARTIST trial. J Clin Oncol. 2012;30(3):268–273. 22184384 10.1200/JCO.2011.39.1953

[36] Shitara K , Van Cutsem E , Bang YJ , Fuchs C , Wyrwicz L , Lee KW , et al. Efficacy and safety of pembrolizumab or pembrolizumab plus chemotherapy vs chemotherapy alone for patients with first‐line, advanced gastric cancer: the KEYNOTE‐062 phase 3 randomized clinical trial. JAMA Oncol. 2020;6(10):1571–1580. 32880601 10.1001/jamaoncol.2020.3370 PMC7489405

[37] Shitara K , Ajani JA , Moehler M , Garrido M , Gallardo C , Shen L , et al. Nivolumab plus chemotherapy or ipilimumab in gastro‐oesophageal cancer. Nature. 2022;603(7903):942–948. 35322232 10.1038/s41586-022-04508-4 PMC8967713

[38] André T , Tougeron D , Piessen G , de la Fouchardière C , Louvet C , Adenis A , et al. Neoadjuvant nivolumab plus ipilimumab and adjuvant nivolumab in localized deficient mismatch repair/microsatellite instability‐high gastric or esophagogastric junction adenocarcinoma: the GERCOR NEONIPIGA phase II study. J Clin Oncol. 2023;41(2):255–265. 35969830 10.1200/JCO.22.00686 PMC9839243

[39] Smyth EC , Verheij M , Allum W , Cunningham D , Cervantes A , Arnold D , et al. Gastric cancer: ESMO clinical practice guidelines for diagnosis, treatment and follow‐up. Ann Oncol. 2016;27(suppl 5):v38–v49. 27664260 10.1093/annonc/mdw350

[40] Japanese Gastric Cancer Association . Japanese gastric cancer treatment guidelines 2018, 5th ed. Gastric Cancer. 2021;24(1):1–21. 32060757 10.1007/s10120-020-01042-y PMC7790804

[41] Yoshida K , Yasufuku I , Terashima M , Young Rha S , Moon Bae J , Li G , et al. International retrospective cohort study of conversion therapy for stage IV gastric cancer 1 (CONVO‐GC‐1). Ann Gastroenterol Surg. 2022;6(2):227–240. 35261948 10.1002/ags3.12515 PMC8889854

[42] Yoshida K , Yamaguchi K , Okumura N , Tanahashi T , Kodera Y . Is conversion therapy possible in stage IV gastric cancer: the proposal of new biological categories of classification. Gastric Cancer. 2016;19(2):329–338. 26643880 10.1007/s10120-015-0575-z PMC4824831

